# Prevalence of smoking and factors associated with smoking cessation among diabetic patients in Chongqing, China: A cross-sectional study

**DOI:** 10.18332/tid/226558

**Published:** 2026-09-09

**Authors:** Heng Luo, Li Zhang, Zhiyong Zhang, Yue Long, Weiping Chen, Changmin Zhang, Fang Liao, Guanglian Zhao

**Affiliations:** 1 School of Nursing, Chongqing Medical University, Chongqing, China; 2 Integrated Traditional Chinese Medicine and Western Medicine Department, Healthcare Center, Chongqing, China; 3 The Second People's Hospital of Dazu District, Chongqing, China; 4 Primary Health Care Centre of Yangjiaping Subdistrict, Chongqing, China; 5 Jiulong Subdistrict Community Health Service Center, Chongqing, China; 6 Shiqiaopu Street Healthcare Center, Chongqing, China

**Keywords:** smoking prevalence, diabetes mellitus, smoking cessation, associated factors

## Abstract

**INTRODUCTION:**

Smoking is associated with accelerated progression of diabetes, whereas smoking cessation supports better disease control. This study aimed to determine the smoking prevalence, factors associated with quitting, and the barriers to and needs for smoking cessation among diabetic patients, in order to provide a basis for targeted smoking cessation interventions.

**METHODS:**

This cross-sectional study was conducted from July to December 2025. A convenience sampling method was used to survey 1736 diabetic patients with a self-administered questionnaire at 10 community health service centers in Chongqing, China. Data were collected on sociodemographic characteristics, glycemic control, history of comorbid chronic conditions, history of diabetic complications, smoke-free home environment, knowledge of the association between smoking and diabetes, and attitudes or beliefs about smoking cessation. Chi-squared tests, Kruskal-Wallis H tests, and multivariate logistic regression were used to identify factors associated with smoking cessation. Adjusted odds ratios (AORs) and 95% confidence intervals (CIs) were calculated for the logistic regression models.

**RESULTS:**

Among the 1736 diabetic patients, 25.6% were current smokers, 16.0% were former smokers, and 58.4% were never smokers. Glycemic control (AOR=0.61; 95% CI: 0.37–1.00), history of comorbid chronic conditions (AOR=3.91; 95% CI: 1.93–7.92), smoke-free home environment (AOR=3.12; 95% CI: 1.99–4.87), knowledge of the association between smoking and diabetes (AOR=1.11; 95% CI: 1.06–1.16), and attitudes or beliefs about smoking cessation (AOR=1.40; 95% CI: 1.31–1.48) were associated with smoking cessation among diabetic patients. Overall, 50.6% of the patients attributed their barriers to cessation to strong nicotine addiction and discomfort after quitting.

**CONCLUSIONS:**

The smoking rate among diabetic patients in Chongqing is relatively high, and fewer than half of current smokers reported an intention to quit smoking. Smoking cessation among diabetic patients was associated with glycemic control, history of comorbid chronic conditions, smoke-free home environment, knowledge of the association between smoking and diabetes, and attitudes or beliefs about smoking cessation.

## INTRODUCTION

Diabetes mellitus (DM) is a group of metabolic disorders characterized by elevated blood glucose (hyperglycemia), predominantly type 2 diabetes mellitus (T2DM), and its prevalence increases significantly with age^1,2^. In 2024, an estimated 589 million adults aged 20–79 years worldwide were living with diabetes, resulting in 3.4 million deaths^2^. China has 148 million diabetic patients, making it the country with the highest number of cases^2^. The pathological changes in diabetes include insulin deficiency, insulin resistance, chronic hyperglycemia, dyslipidemia, and disturbances in other metabolic pathways, leading to simultaneous abnormalities in both vascular and parenchymal tissues, which are the primary causes of chronic diabetic complications^3^. These complications, such as diabetic neuropathy, diabetic nephropathy, and diabetic cardiovascular disease, not only severely reduce the quality of life of diabetic patients but also increase their risk of death^4^.

Smoking poses a dual burden to the health of diabetic patients by directly damaging their organs and by reducing the efficacy of antidiabetic medications. Studies have reported that nicotine in tobacco increases the risk of developing diabetes through insulin resistance, metabolic disorders, and inhibition of insulin signaling pathways^5,6^. Simultaneously, harmful substances in tobacco act on the vascular endothelium, exacerbating and accelerating vascular damage, thereby increasing the risk of diabetic microvascular complications^7,8^. Compounds in tobacco smoke, primarily polycyclic aromatic hydrocarbons (PAHs), may also alter the metabolism of certain oral antidiabetic drugs by inducing or inhibiting drug-metabolizing enzymes^9^. This may reduce drug efficacy or produce toxic effects and may in turn affect glycemic control and contribute to other adverse reactions^9^. Diabetic patients who smoke face the combined burden of tobacco and the effects of diabetes itself on blood vessels and nerves, and previous studies have reported higher rates of complications and mortality in smokers than in non-smokers. A meta-analysis of 89 prospective cohort studies reported that diabetic patients who smoke had an approximately 50% higher risk of death and adverse cardiovascular events, such as coronary heart disease, stroke, and heart failure, than non-smokers^10^. The risk of lower-extremity amputation related to diabetic foot was reported to be 1.65 times higher^11^.

Smoking behavior remains prevalent among the diabetic population. A 2019 report on the global prevalence of tobacco use in type 2 diabetes mellitus indicated that one in five smokers had diabetes^12^. In 2022, Durlach et al.^13^ reported that approximately 20% of people with type 2 diabetes and 30% of those with type 1 diabetes smoke. To reduce the harm of tobacco to diabetic patients, the American Diabetes Association (ADA) and the International Diabetes Federation strongly recommend smoking cessation as an essential component of diabetes management^2,14^. Smoking cessation interventions for diabetic patients have been widely implemented, but their effectiveness varies. In 2023, researchers in Malaysia incorporated smoking cessation support into routine practice by adding a 5-minute brief smoking cessation consultation provided by physicians to standard diabetes care; however, the results showed no improvement in glycemic control or smoking cessation rates among diabetic patients who smoked^15^. Similarly, a smoking cessation intervention study in Hong Kong reported that brief counseling based on stage-matched behavioral change, together with a diabetes-specific smoking cessation booklet provided by nurses, was not associated with reduced smoking or improved glycemic control in diabetic patients^16^. A common feature of these less effective interventions is the use of standardized brief advice for smokers, which overlooks the complex individual differences and specific cessation needs of patients. By contrast, a study in Taiwan conducted a targeted survey before the intervention and explored diabetic patients’ smoking cessation motives and barriers in depth. By tailoring health guidance and pharmacotherapy regimens to patients’ levels of nicotine dependence, disease awareness, and social environment, that study reported a significantly higher smoking cessation rate^17^.

China has implemented a series of policies and measures to control the tobacco epidemic and reduce the health hazards of tobacco, but less attention has been paid to smoking cessation among diabetic patients^18^. Chongqing is the largest municipality in China, with a population of over 30 million^19^. In 2024, the smoking rate among the population aged >15 years in Chongqing was 22.59%, while the prevalence of diabetes was 10.37%^19,20^. The current prevalence of smoking among diabetic patients in Chongqing remains unclear. Therefore, this study aimed to investigate the smoking prevalence and the factors associated with smoking cessation among diabetic patients in Chongqing, to identify their willingness to quit and the barriers to smoking cessation.

## METHODS

### Study setting and participants

This was a cross-sectional study conducted from July to December 2025. A convenience sampling method was used to recruit diabetic patients from 10 community health service centers located in the eastern, western, northern, southern, and central regions of Chongqing. The inclusion criteria were that participants: 1) met the diagnostic criteria for diabetes according to the Chinese Guidelines for the Prevention and Treatment of Diabetes (2024 Edition); 2) had good communication and comprehension ability; and 3) were willing to participate in this study and provide informed consent. Participants were excluded if they had severe mental disorders or cognitive impairment that prevented them from understanding the study content or providing informed consent.

Trained investigators explained the purpose of the study to participants. After obtaining informed consent, participants with smartphones could choose to complete an electronic questionnaire by scanning a QR code, while those without smartphones completed a paper questionnaire. The collected paper questionnaires were entered into a database independently by two researchers, and the entries were verified for consistency before use. The required sample size was calculated^13^ based on a smoking rate of 20% (p=0.20) among diabetic patients. With the permissible error δ set at 0.05 and the significance level α=0.05, and allowing for 10% incomplete responses, the minimum required sample size was 274. A total of 1800 questionnaires were collected, comprising 1000 paper questionnaires and 800 electronic questionnaires. After excluding 30 incomplete paper questionnaires and 34 electronic questionnaires completed in <120 seconds, 1736 valid questionnaires were included in the analysis.

This study was approved by the Research Ethics Committee of Chongqing Medical University (Approval number: 2025-035; Date: 30 May 2025).

### Definitions of outcomes

In this study, current smokers were defined as individuals who had smoked continuously or cumulatively for ≥6 months and were still smoking at the time of the study; former smokers were defined as individuals who had smoked in the past but had not smoked within the last 28 days; never smokers were defined as individuals who had never smoked in their lifetime or had smoked <100 cigarettes^21,22^.

### Measures and questionnaire

The questionnaire was developed based on the literature^10,21,23^ and the knowledge-attitude-belief-practice (KABP) model. Experts in tobacco medicine, clinical smoking cessation, epidemiology and biostatistics, health behavior, and community nursing were invited to revise and refine the questionnaire content through two rounds of expert consultation. Fifty diabetic patients who met the inclusion and exclusion criteria participated in the pilot and pretest of the questionnaire to assess its comprehensibility and verify its validity. The questionnaire consisted of four sections:

*Sociodemographic characteristics*

These included age, gender (male, female), education level (primary school or lower, junior high school, high school/vocational/technical, college or higher), marital status (married, unmarried/other), occupation(worker/business/service, self-employed/private business, administrative/technical/management, unemployed or awaiting employment, other), glycemic control (controlled, uncontrolled), history of comorbid chronic conditions (1, 2, or ≥3), history of diabetic complications(0, 1, 2, or ≥3), body mass index (BMI, kg/m^2^), smoke-free home environment (Yes, No). Age, education level, marital status, occupation, history of comorbid chronic conditions, and BMI were considered potential confounders, *a priori*.

*Knowledge of the association between smoking and diabetes*

This section comprised 15 items grouped into four domains.

Harms of smoking: 1) smoking can cause type 2 diabetes; 2) smoking increases the risk of diabetic complications; 3) the greater the amount and the longer the duration of smoking, the higher the risk of type 2 diabetes; 4) secondhand smoke exposure increases the risk of diabetes; and 5) nicotine addiction is a chronic disease’.

Benefits of smoking cessation: 1) smoking cessation helps with glycemic control; 2) smoking cessation reduces the incidence and mortality of type 2 diabetes; and 3) the benefits of smoking cessation cannot be replaced by a reasonable diet and exercise.

Effectiveness of smoking cessation services: 1) Smoking cessation clinics are an effective model for quitting; and 2) Scientific smoking cessation methods include consulting a doctor, using cessation medications, and calling a quitline.

Awareness of five smoking cessation resources: 1) cessation medications, 2) cessation websites, 3) cessation apps, 4) cessation clinics, and 5) quitlines.

Knowledge of the association between smoking and diabetes was assessed using a 5-point Likert scale ranging from strongly disagree to strongly agree (scores 1–5). The theoretical median was 3; a score of ≤3 on each item was defined as low knowledge, and a score >3 as high knowledge. Awareness of cessation resources was measured on a 5-point Likert scale (1=not at all aware, 2=slightly aware, 3=familiar, 4=very familiar but never used, 5=used). Total scores ranged from 5 to 25. Based on the percentile distribution and the observed clustering of the data, awareness was categorized into three groups: 5 points (unaware), 6–10 points (moderate), and ≥11 points (aware). The Cronbach’s α for this section was 0.885, indicating good reliability.

*Attitudes and beliefs about smoking cessation among diabetic patients*

These were assessed with 10 items covering common perceptions of smoking (seven items: smoking relieves hunger, helps with emotional control, keeps the mind clear, reduces stress, controls weight, and relates to personal preference) and self-efficacy for smoking cessation (three items). Responses were rated on a 5-point Likert scale from strongly disagree to strongly agree. Items 1–7 were negatively phrased and reverse-scored, so that higher scores indicated stronger disagreement with the negative statement, while items 8–10 were positively phrased, so that higher scores indicated stronger cessation beliefs. The maximum total score was 50 points, with higher scores indicating stronger cessation beliefs. The Cronbach’s α for this section was 0.770.

*Barriers to smoking cessation*

For diabetic patients who smoke, the questionnaire also assessed their barriers to smoking cessation (including nicotine dependence level, tobacco withdrawal symptoms, and self-reported major barriers to quitting) and cessation needs (including preferred forms of assistance, desired health education content, and expected types of support providers).

### Statistical analysis

Data were analyzed using IBM SPSS Statistics (version 27.0). Qualitative data are presented as frequencies and percentages. Quantitative data with non-normal distribution are presented as medians with interquartile range (IQR), and normality was assessed using the Shapiro-Wilk test. Group differences in qualitative data were examined using the chi-squared test, while differences in quantitative data were analyzed using the Kruskal-Wallis H test. The Bonferroni method was applied to adjust significance levels for multiple comparisons to identify pairwise differences among the three groups (never smokers, current smokers, and former smokers). Comparisons of cessation attitudes and beliefs between current smokers and former smokers were performed using the Mann-Whitney U test. Independent variables that were statistically significant in the univariate analyses were included in collinearity diagnostics, and all variance inflation factor (VIF) values were <5. Two separate multivariable binary logistic regression analyses were performed with smoking status among male participants (current smokers=1, never smokers=0) and smoking cessation status (former smokers=1, current smokers=0) as dependent variables, respectively. Variables that were statistically significant in univariate analyses (p<0.05) were entered as independent variables. The *never smokers versus current smokers* model included 10 independent variables (age, education level, marital status, occupation, glycemic control, history of comorbid chronic conditions, BMI, smoke-free home environment, knowledge of the association between smoking and diabetes, awareness of cessation resources), and the *former smokers versus current smokers* model included 11 independent variables (the same 10 variables as the *never smokers vs current smokers* model, plus attitudes/beliefs score). Twotailed tests were used, and p<0.05 was considered statistically significant for all analyses.

## RESULTS

### Study sample characteristics

A total of 1736 diabetic patients were surveyed in this study, with a median age of 62 years (IQR: 56–70). There were 914 males (52.6%) and 822 females (47.4%). Among them, 1013 (58.4%) were never smokers, 445 (25.6%) were current smokers, and 278 (16.0%) were former smokers. Table 1 shows that the differences in baseline characteristics among diabetic patients with different smoking statuses were statistically significant (p<0.05).

**Table 1:** Comparison of baseline characteristics of diabetic patients stratified by smoking status, Chongqing, China, 2025 (N=1736)

*Characteristics*	*Total (N=1736) n (%)*	*Never smokers ^a^ (N=1013) n (%)*	*Current smokers ^b^ (N=445) n (%)*	*Former smokers ^c^ (N=278) n (%)*	*χ^2^/H*	*p*	*Bonferroni*
**Age** (years), median (IQR)	62 (56–70)	66 (58–72)	62 (55–69)	67.5 (60–73)	70.374	**<0.001**	b<a<c
**Gender**					1000.208	**<0.001**	
Male	914 (52.6)	209 (20.6)	434 (97.5)	271 (97.5)			a<b, c
Female	822 (47.4)	804 (79.4)	11 (2.5)	7 (2.5)			
**Education level**					47.685	**<0.001**	
Primary school or lower	719 (41.4)	484 (47.8)	137 (30.8)	98 (35.3)			
Junior high school	620 (35.7)	316 (31.2)	188 (42.2)	116 (41.7)			
High school/vocational/technical	319 (18.4)	163 (16.1)	101 (22.7)	55 (19.8)			
College or higher	78 (4.5)	50 (4.9)	19 (4.3)	9 (3.2)			
**Marital status**					16.146	**<0.001**	
Married	1525 (87.8)	864 (85.3)	402 (90.3)	259 (93.2)			a<b, c
Unmarried/other	211 (12.2)	149 (14.7)	43 (9.7)	19 (6.8)			
**Occupation**					175.269	**<0.001**	
Worker/business/service	638 (36.8)	308 (30.4)	199 (44.7)	131 (47.1)			
Selfemployed/private business	240 (13.8)	110 (10.9)	101 (22.7)	29 (10.4)			
Administrative/technical/management	183 (10.5)	89 (8.8)	57 (12.8)	37 (13.3)			
Unemployed/awaiting employment	319 (18.4)	233 (23.0)	66 (14.8)	20 (7.2)			
Other	356 (20.5)	273 (26.9)	22 (4.9)	61 (21.9)			
**Glycemic control**					14.628	**<0.001**	
Controlled	1195 (68.8)	701 (69.2)	281 (63.1)	213 (76.6)			c>a, b
Uncontrolled	541 (31.2)	312 (30.8)	164 (36.9)	65 (23.4)			
**History of comorbid chronic conditions**					63.991	**<0.001**	
1	754 (43.4)	404 (39.9)	252 (56.6)				
2	616 (35.5)	354 (34.9)	154 (34.6)				
≥3	366 (21.1)	255 (25.2)	39 (8.8)				b<a, c
**History of diabetic complications**					9.406	**0.009**	
0	1460 (84.1)	839 (82.8)	393 (88.3)				
1	167 (9.6)	102 (10.1)	40 (9.0)				
2	56 (3.2)	35 (3.5)	9 (2.0)				
≥3	53 (3.1)	37 (3.7)	3 (0.7)				b<a, c
**BMI** (kg/m²), median (IQR)	24.0 (22.5–25.9)	24.0 (22.1–25. 9)	23.9 (23.0–25.8)	24.5 (23.2–26.3)	11.655	**0.003**	a, b<c
**Smoke-free home environment**					106.103	**<0.001**	
No	791 (45.6)	417 (41.2)	291 (65.4)	83 (29.9)			
Yes	945 (54.4)	596 (58.8)	154 (34.6)	195 (70.1)			b<a<c

Bonferroni correction: a) never smokers; b) current smokers; c) former smokers. P-values were calculated using the chi-squared test or the Kruskal-Wallis H test. IQR: interquartile range. BMI: body mass index.

Further pairwise comparisons revealed that current smokers were predominantly male and younger than the other two groups. The rate of achieving glycemic control among current smokers (63.1%) was lower than that of never smokers (69.2%) and former smokers (76.6%), whereas their proportions of having multiple comorbid chronic conditions and diabetic complications were lower than those of never smokers and former smokers. Former smokers had a median BMI of 24.5 kg/m^2^, classified as overweight, which was higher than that of never smokers (24.0) and current smokers (23.9). Current smokers had the lowest proportion of having a smoke-free home environment (34.6%), while former smokers had the highest proportion (70.1%). These differences were statistically significant (p<0.05).

There were significant differences in the level of knowledge of the association between smoking and diabetes among diabetic patients with different smoking behaviors (p<0.05). The proportion of never smokers with high scores in the two domains of smoking harms (68.0%) and benefits of smoking cessation (71.0%) was higher than that in the other two groups of diabetic patients. The proportion of never smokers with high scores in the effectiveness of smoking cessation services (61.9%) was higher than that of current smokers (52.1%). The level of awareness of smoking cessation resources among current smokers (7.4%) was higher than that among never smokers (1.3%), and the difference was statistically significant (p<0.05) (Table 2).

**Table 2:** Knowledge of the association between smoking and diabetes, and awareness of smoking cessation resources among diabetic patients stratified by smoking status, Chongqing, China, 2025 (N=1736)

	*Total (N=1736) n (%)*	*Never smokers ^a^ (N=1013) n (%)*	*Current smokers ^b^ (N=445) n (%)*	*Former smokers ^c^ (N=278) n (%)*	*χ^2^/H*	*p*	*Bonferroni*
**Harms of smoking**					13.961	**<0.001**	
Low score (5–15)	618 (35.6)	324 (32.0)	179 (40.2)	115 (41.4)			
High score (16–25)	1118 (64.4)	689 (68.0)	266 (59.8)	163 (58.6)			a>b, c
**Benefits of smoking cessation**					25.511	**<0.001**	
Low score (3–9)	587 (33.8)	294 (29.0)	185 (41.6)	108 (38.8)			
High score (10–15)	1149 (66.2)	719 (71.0)	260 (58.4)	170 (61.2)			a>b, c
**Effectiveness of cessation services**					13.085	**<0.001**	
Low score (2–6)	722 (41.6)	386 (38.1)	213 (47.9)	123 (44.2)			
High score (7–10)	1014 (58.4)	627 (61.9)	232 (52.1)	155 (55.8)			a>b
**Total knowledge score, median** (IQR)	34 (30–38)	36 (32–39)	33 (30–36)	34 (30–39)	46.930	**<0.001**	a>c>b
**Awareness of cessation resources**					42.526	**<0.001**	
Unaware	1019 (58.7)	641 (63.3)	208 (46.7)	170 (61.2)			
Moderate	652 (37.6)	359 (35.4)	204 (45.8)	89 (32.0)			
Aware	65 (3.7)	13 (1.3)	33 (7.4)	19 (6.8)			a<b, c

Bonferroni correction: a) never smokers; b) current smokers; c) former smokers. P-values were calculated using the chi-squared test or the Kruskal-Wallis H test. IQR: interquartile range.

The total score of smoking cessation attitudes and beliefs was significantly higher in former smokers than in current smokers (p<0.001), as shown in Table 3. Diabetic patients who had quit smoking (median: 4) disagreed more strongly than current smokers (median: 3) with the statements: ‘Smoking relieves hunger’, ‘Diabetes has already made me give up many things I like, so I do not want to quit smoking’, ‘I am afraid that weight gain after quitting will lead to poor disease control’, ‘Quitting smoking takes time and energy, and I currently do not have the energy to cope’, and ‘Smoking can relieve stress, and I feel I cannot find an alternative after quitting’ (p<0.001). There was a statistically significant difference between the two groups regarding the statement ‘Smoking often helps me control my emotions’ (p=0.006), whereas no statistically significant difference was found for the statement ‘Smoking keeps my mind clear’ (p=0.608).

**Table 3:** Smoking cessation attitudes and beliefs among diabetic patients by smoking status, Chongqing, China, 2025 (N=723)

*Items*	*Current smokers (N=445) Median (IQR)*	*Former smokers (N=278) Median (IQR)*	*p*
Smoking relieves hunger	3 (2–4)	4 (3–4)	**<0.001**
Smoking often helps me control my emotions	2 (2–3)	2 (2–4)	**0.006**
Smoking keeps my mind clear	2 (2–3)	2 (2–3)	0.608
Diabetes has already made me give up many things I like, so I do not want to quit smoking	3 (2–4)	4 (3–4)	**<0.001**
I am afraid that weight gain after quitting will lead to poor disease control	3 (2–4)	4 (3–4)	**<0.001**
Quitting smoking takes time and energy, and I currently do not have the energy to cope	3 (2–4)	4 (4–4)	**<0.001**
Smoking can relieve stress, and I feel I cannot find an alternative after quitting	3 (2–3)	4 (3–4)	**<0.001**
If I have family support for quitting, I will be more confident to overcome the disease	3 (3–4)	4 (3–4)	**<0.001**
Once I decide to quit smoking, I will succeed	3 (2–4)	4 (4–5)	**<0.001**
Because there are successful role models around me, I can quit successfully like them	3 (3–4)	4 (3–4)	**<0.001**
Total belief score, median (IQR)	28 (26–31)	35 (31–38)	**<0.001**

Participants’ smoking cessation attitudes and beliefs were assessed using a Likert scale, with responses scored from 1 (strongly disagree) to 5 (strongly agree). Items 1–7 were reverse-scored and items 8–10 were positively scored. P-values were calculated using the Mann-Whitney U test. IQR: interquartile range.

Former smokers (median: 4) more strongly endorsed the role of family support, successful role models for smoking cessation, and self-efficacy in increasing confidence to quit, and had higher smoking cessation self-efficacy compared to current smokers (median: 3) (p<0.001).

The binary logistic regression analyses were restricted to male participants due to the extremely small number of female smokers (n=11; 2.5%) in the sample (Table 4).

**Table 4:** Multivariable analysis of smoking status and associated factors among male diabetic patients, Chongqing, China, 2025 (N=914)

*Variables*	*Never smokers vs current smokers*			*Former smokers vs current smokers*		
	*β*	*p*	*AOR (95 % CI)*	*β*	*p*	*AOR (95 % CI)*
**Education level**						
Primary school or lower (ref.)			1			1
Junior high school	0.18	0.491	1.19 (0.72–1.96)	-0.28	0.320	0.76 (0.44–1.31)
High school/vocational/technical	0.07	0.817	1.08 (0.58–1.98)	-0.35	0.323	0.71 (0.36–1.40)
College or higher	-1.22	**0.006**	0.30 (0.12–0.71)	-0.76	0.235	0.47 (0.13–1.64)
**Glycemic control**						
Controlled (ref.)			1			1
Uncontrolled	0.18	0.400	1.20 (0.78–1.85)	-0.50	**0.048**	0.61 (0.37–1.00)
**History of comorbid chronic conditions**						
1 (ref.)			1			1
2	-0.09	0.718	0.92 (0.58–1.46)	0.24	0.351	1.27 (0.77–2.10)
≥3	-0.51	0.139	0.60 (0.31–1.18)	1.36	**<0.001**	3.91 (1.93–7.92)
**Smoke-free home environment**						
No (ref.)			1			1
Yes	-1.28	**<0.001**	0.28 (0.19–0.42)	1.14	**<0.001**	3.12 (1.99–4.87)
**Knowledge score**	-0.10	**<0.001**	0.91 (0.87–0.94)	0.10	**<0.001**	1.11 (1.06–1.16)
**Attitudes/beliefs score**				0.33	**<0.001**	1.40 (1.31–1.48)

AOR: adjusted odds ratio.

### Multivariate analysis results for never smoking versus current smoking

Compared with those with primary school education or lower, having a university education was negatively associated with current smoking (AOR=0.30; 95% CI: 0.12–0.71). A smoke-free home environment, compared with a home environment where smoking occurred, was negatively associated with current smoking (AOR=0.28; 95% CI: 0.19–0.42). In addition, higher knowledge scores regarding the association between smoking and diabetes were negatively associated with the likelihood of current smoking (AOR=0.91; 95% CI: 0.87–0.94).

### Multivariate analysis results for former smoking versus current smoking

Poor glycemic control was negatively associated with smoking cessation (AOR=0.61; 95% CI: 0.37–1.00). Having ≥3 comorbid chronic conditions was positively associated with smoking cessation (AOR=3.91; 95% CI: 1.93–7.92). A smoke-free home environment, compared with a home environment where smoking occurred, was positively associated with smoking cessation (AOR=3.12; 95% CI: 1.99–4.87). Higher knowledge scores were positively associated with the likelihood of smoking cessation (AOR=1.11; 95% CI: 1.06–1.16). Higher attitudes or beliefs scores were positively associated with the likelihood of smoking cessation (AOR=1.40; 95% CI: 1.31–1.48).

### Nicotine dependence, smoking cessation intention, and cessation needs among diabetic patients who smoke

Among the 445 diabetic patients who smoked, more than half (56.4%) had moderate or severe nicotine dependence, and 404 (91.8%) experienced at least one tobacco withdrawal symptom when attempting to quit or reduce smoking. Only 199 (44.7%) of these patients had an intention to quit smoking. Regarding barriers to cessation, 50.6% of the patients attributed it to strong nicotine addiction and discomfort after quitting.

In terms of cessation needs, 119 (59.8%) chose smoking cessation medications, and 114 (57.3%) chose smoking cessation clinics. Regarding health education content, patients reported high needs for information on diabetes management (66.8%), glycemic control (59.3%), and coping skills for smoking cessation discomfort (51.3%). Regarding sources of help, healthcare professionals and family members were each selected by 116 (58.3%) respondents.

## DISCUSSION

This study found that the prevalence of current smoking among diabetic patients in Chongqing was higher compared to the local general adult population (22.59%) and diabetic patients in Ningbo (21.6%)^19,24^; however, the prevalence of former smokers among diabetic patients was lower compared to the 29.9% observed among diabetic patients across Europe^25^. This suggests that particular attention should be given to smoking cessation guidance and interventions for this population. In this study, lower knowledge of the association between smoking and diabetes, more negative attitudes, lower self-efficacy, and a greater number of cessation barriers were associated with smoking among diabetic patients.

The present study found that smoking behavior was more common among male diabetic patients with a lower education level. This may be related to the national context in China, where the smoking rate among males is significantly higher than that among females^26^. Education level, as a commonly used proxy indicator of socioeconomic status, showed a negative association with smoking behavior, which is consistent with the findings of a survey covering 82 low- and middle-income countries, suggesting an inverse relationship between socioeconomic status and smoking behavior^27^. Furthermore, poor glycemic control observed in this population may be related to nicotine-induced insulin resistance, delayed insulin absorption, and impaired β-cell function, which are also among the causes of complications^6^. This study showed that diabetic smokers had low awareness that smoking causes diabetes and exacerbates and accelerates its complications, and lacked knowledge related to smoking cessation; this is consistent with the qualitative findings reported by Grech et al.^28^ in the diabetic population and may be associated with lower health literacy among smokers themselves. Furthermore, the low awareness of smoking cessation resources and underutilized professional cessation support observed in this study, aligns with the finding from the 2020 National Adult Tobacco Survey (NATS) that 93.1% of smokers used unassisted smoking cessation^29^.

Lack of knowledge about the association between smoking and diabetes may prevent patients from forming correct attitudes toward smoking cessation. In this study, diabetic patients who smoked showed higher agreement with the notions that smoking relieves hunger, controls emotions, and reduces stress, which may be related to the physiological effects of nicotine in transiently suppressing appetite and alleviating withdrawal symptoms^30^. Their reported willingness to quit smoking was low. Smokers also showed a stronger tendency toward delay discounting, whereby the immediate gratification of smoking may lead patients to discount its delayed harms^31^. In this study, older former smokers had a higher prevalence of multiple comorbid chronic conditions and complications than current smokers. This pattern may reflect the cumulative nature of tobacco-related harm over time, and the possibility that the presence of multiple chronic diseases and complications is associated with the adoption of health behaviors such as smoking cessation; however, the cross-sectional design does not allow this temporal sequence to be confirmed. Young diabetic smokers in the early stages of disease, who have nicotine dependence and poor glycemic control but have not yet developed severe complications, may represent an important window for behavioral intervention. This may be particularly relevant for those with a lower level of education, who tend to have weaker awareness of the harms of smoking and to use smoking for stress relief.

This study found that nicotine dependence and withdrawal symptoms were barriers commonly reported by patients, which is consistent with previous literature^28,32^. The study also found that former smokers had a higher BMI than current smokers, which aligns with evidence on post-smoking-cessation weight gain (PSCWG) globally and may be related to the cessation of nicotine’s central nervous system effects following smoking cessation, increased appetite, and decreased energy expenditure^30,33^. PSCWG may temporarily exacerbate diabetes by impairing glycemic control and is one of the major barriers to smoking cessation among diabetic patients^34^. However, cohort studies have demonstrated that even with increased BMI, the overall mortality risk of quitters remains significantly lower than that of continuing smokers, and preventing excessive weight gain can maximize the benefits of smoking cessation in controlling diabetic complications^35,36^. Family support has been associated with greater success in smoking cessation^32^. In this study, smokers commonly expressed a desire for support from both professionals and family members.

### Limitations

Several limitations of this study should be acknowledged. First, smoking/cessation status and former smokers’ history (e.g. daily cigarette consumption and years of smoking) relied on self-report, without biochemical verification (e.g. urinary cotinine testing). Given the social stigma of smoking in China, patients may underreport smoking behavior, leading to misclassification of smoking status and thus underestimation of the smoking rate. Additionally, retrospective recall among former smokers may introduce information bias, which affects the accuracy of nicotine dependence assessment. Second, this study surveyed diabetic patients only in Chongqing; because smoking behavior and tobacco control policies vary considerably by region, caution is needed when generalizing the findings to the whole country. Third, despite adjusting for multiple potential confounders in the multivariable models, residual confounding from unmeasured or inadequately measured variables cannot be entirely ruled out. Fourth, the cross-sectional design cannot establish causal relationships between variables. Future prospective cohort studies are needed to explore these issues further.

## CONCLUSIONS

This study found that the smoking rate among diabetic patients in Chongqing was high, but that the proportion of current smokers with quit intentions was low. Glycemic control, history of comorbid chronic conditions, smoke-free home environment, knowledge of the association between smoking and diabetes, and attitudes or beliefs about smoking cessation were associated with smoking cessation among diabetic patients. Concerns about weight gain, nicotine dependence, and withdrawal symptoms were the main reported barriers to cessation.

## Data Availability

The data supporting this research are available from the authors on reasonable request.
